# Visually Evoked Response Differences to Contrast and Motion in Children with Autism Spectrum Disorder

**DOI:** 10.3390/brainsci8090160

**Published:** 2018-08-24

**Authors:** Lauren C. Shuffrey, Lisa Levinson, Alexis Becerra, Grace Pak, Dayna Moya Sepulveda, Alicia K. Montgomery, Heather L. Green, Karen Froud

**Affiliations:** 1 Department of Biobehavioral Sciences, Teachers College, Columbia University 525 W 120th Street, New York, NY 10027, USA; lml2155@tc.columbia.edu (L.L.); ab4211@tc.columbia.edu (A.B.); grey.pak@gmail.com (G.P.); damoya@uc.cl (D.M.S.); greenhl@email.chop.edu (H.L.G.); kfroud@tc.columbia.edu (K.F.); 2 Department of Child and Adolescent Psychiatry, Columbia University Medical Center/New York State Psychiatric Institute, 1051 Riverside Drive, New York, NY 10032, USA; alicia.k.montgomery@gmail.com; 3 Center for Autism and the Developing Brain, New York-Presbyterian Hospital, 21 Bloomingdale Road, White Plains, NY 10605, USA; 4 Department of Education, Pontificia Universidad Católica de Chile, Villarrica, La Araucanía Region 4930000, Chile; 5 Sydney Children’s Hospital, Community Health Center, High Street, Randwick NSW 2031, Australia; 6 Department of Radiology, Children’s Hospital of Philadelphia, 3401 Civic Center Blvd, Philadelphia, PA 19104, USA

**Keywords:** autism, electroencephalography, P1 event-related potential, visual contrast, visual motion

## Abstract

High-density electroencephalography (EEG) was used to examine the utility of the P1 event-related potential (ERP) as a marker of visual motion sensitivity to luminance defined low-spatial frequency drifting gratings in 16 children with autism and 16 neurotypical children. Children with autism displayed enhanced sensitivity to large, high-contrast low-spatial frequency stimuli as indexed by significantly shorter P1 response latencies to large vs. small gratings. The current study also found that children with autism had larger amplitude responses to large gratings irrespective of contrast. A linear regression established that P1 adaptive mean amplitude for large, high-contrast sinusoidal gratings significantly predicted hyperresponsiveness item mean scores on the Sensory Experiences Questionnaire for children with autism, but not for neurotypical children. We conclude that children with autism have differences in the mechanisms that underlie low-level visual processing potentially related to altered visual spatial suppression or contrast gain control.

## 1. Introduction

Autism spectrum disorder (ASD) is a neurodevelopmental disorder characterized by impairments in social interaction, communication, and the presence of stereotypic behaviors or restricted interests [1]. One of the diagnostic criteria set forth by the DSM-5 includes the presence of hyper- or hypo-reactivity to sensory input(s), or unusual interests in sensory input(s) [1]. This may include a heightened or attenuated response to temperature, auditory input, texture, smell, visual input, or movement. It is estimated that between 42% and 88% of individuals with ASD have some impairment in sensory processing [2].

Visual sensory differences are perhaps the most widely researched sensory issue in ASD, with abnormalities in multiple domains having been documented. Observed visual sensory differences in ASD include (but are not limited to): deficits in low-level visual processing; atypical motion perception; abnormal perceptual integration; holistic processing deficits; reduced sensitivity to biological motion; enhanced perception of fine details; impairments in object-boundary detection; and deficits in facial processing [3,4,5,6]. Differences in low-level visual processing, which is considered the foundation for higher-order visual perception, can disrupt downstream processes such as visual attention to social stimuli, facial processing, and multi-sensory integration [7,8]. There is also evidence to suggest that developmental impairments in the building blocks of higher-order sensory perception can affect both social interaction and communication [8].

Low-level visual processing in autism has been investigated using a wide range of paradigms with many studies employing psychophysical behavioral paradigms that measure motion discrimination as behavioral duration response thresholds to drifting grating stimuli of varying stimulus speed, size, and/or contrast. Results from these studies have been highly inconsistent, even when very similar methodology was utilized [9]. Prior studies have shown: (a) no significant differences in contrast sensitivity between adolescents with autism and controls [10]; (b) more-able children with autism demonstrated decreased motion discrimination thresholds (i.e., faster behavioral responses) to high-contrast drifting gratings in all stimulus sizes compared to controls [11]; (c) children with a wide range of intellectual abilities demonstrated no motion discrimination threshold differences to high-contrast gratings, but showed significantly delayed processing of small stimuli in both high- and low-contrast conditions compared to the neurotypical group [12]; (d) more-able children and adolescents with autism demonstrated longer motion discrimination thresholds to small stimuli (1°) regardless of stimulus contrast [13].

Although prior study results have been mixed, each suggests spatial suppression and/or contrast gain control may be altered in individuals with autism. Efficient visual perception requires the brain to filter irrelevant information by various mechanisms of suppression [14]. The receptive field of an individual sensory neuron is the region within the visual field in which a stimulus will trigger activation of that neuron, resulting in an action potential. However, this response is modulated by stimuli arising from the surround, or non-classical receptive field [15]. Spatial suppression, also known as surround suppression, is a visual-sensory effect that decreases neuronal reactivity to stimuli outside of a neuron’s classic receptive field, and is both size and contrast dependent. Behaviorally, this effect manifests in neurotypical populations in tasks of directional motion perception, so that as stimulus size increases the motion direction of high-contrast patterns becomes increasingly difficult to perceive [16].

The suppression of large, high-contrast stimuli in the surround may improve the saliency and clarity of smaller moving objects in the center of the classic receptive field [17]. Neurons in the primary visual cortex are size-selective, such that a neuronal response rate increases until a preferred size is reached, and once both the classic receptive field and surround are stimulated, the magnitude of response is attenuated [18]. Spatial suppression observed in V1 is likely reflective of modulatory mechanisms operating at varying levels of the visual system such as in retinal ganglion cells, the lateral geniculate nucleus, the cortex, and cortical receptive fields [18,19]. Cortical processing of responses to visual stimuli involves modulation via feedforward and negative feedback mechanisms, which are thought to play a role in attention and visual awareness [20].

The magnitude of the suppressive response is contrast-dependent, with the greatest degree of suppression occurring in conditions of high contrast and reduced or absent suppression occurring in conditions of low contrast [21,22]. The lack of suppression in conditions of low contrast can be explained by the inhibitory mechanism known as contrast gain control, which describes the tendency for the majority of neurons in the visual cortex to increase their firing rates with increasing contrast levels [23]. Gain control allows for sensory systems to adapt responses dependent on the context of the stimuli, and can be influenced by neural excitation, neural inhibition, and feedback connections [24].

Although prior motion perception studies have relied on duration thresholds in order to investigate motion perception in autism, behavioral threshold measures require a degree of participant comprehension and cooperation that may be beyond the capability of younger pediatric populations, and of some populations with developmental disorders. In contrast to perceptual discrimination threshold responses, event-related potentials (ERPs) derived from electroencephalographic (EEG) recordings provide a non-invasive electrophysiological method to gain objective insights regarding the integrity of the visual system. The visual P1 ERP can be recorded from the scalp over occipital electrode sites in response to a pre-attentional visual stimulus, and does not rely on participant behavioral responses. Against this background, the purpose of this research is to determine whether P1 ERP latency and amplitude can be used to examine motion sensitivity to luminance defined low-spatial frequency drifting gratings in children with autism compared to control participants. If children with autism have differences in spatial suppression, we would expect them to display enhanced sensitivity to large, high-contrast gratings reflected by shorter P1 ERP latency. In order to assess if prior observed behavioral differences are primarily affecting suppression or also affecting contrast gain control, we also investigated motion sensitivity in conditions of low contrast.

## 2. Methods

### 2.1. Assessments

All participants were administered the following evaluations: Visual acuity screening; the Pelli-Robson Contrast Sensitivity Chart [25]; Stanford-Binet Intelligence Scales, Fifth Edition Abbreviated IQ (SB5 ABIQ) [26]; and the Childhood Autism Rating Scale, Second Edition (CARS-2) [27]. The experimental group was administered the Autism Diagnostic Observation Schedule, Second Edition (ADOS-2) [28]. Comparison participants were screened for autism using the CARS-2. All parents of participants completed the Childhood Autism Rating Scale, Second Edition (CARS-2) Parent Questionnaire [27]; the Sensory Experiences Questionnaire [29]; a Family Medical/Psychiatric History Questionnaire; and a Demographic Questionnaire.

### 2.2. Participants

Forty children aged 6 to 12 years, 21 with a diagnosis of autism spectrum disorder (ASD) and 19 neurotypical (NT) comparison children, were recruited to participate. Eight participants were excluded from the present study due to: unwillingness to wear the EEG net (*n* = 2), participant movement resulting in too few usable trials per condition (*n* = 4), abbreviated IQ < 75 (*n* = 1), and current use of psychiatric medication (*n* = 1). After exclusions, 32 participants were included in data analysis, 16 with a diagnosis of ASD and 16 NT comparison children. Participants were included from diverse U.S. racial and ethnic minority groups. Although the ASD group (mean age = 8.04 years, SD = 1.78) was younger on average than the NT group (mean age = 9.44 years, SD = 2.29), this difference was not statistically significant (t (30) = −1.92, *p* = 0.06). All participants reported no history of neurological disorder, and had normal or corrected-to-normal vision as indicated by the Snellen Eye Chart. Additionally, all participants had normal contrast sensitivity function, as indicated by the Pelli-Robson Contrast Sensitivity Chart. No participants had used psychiatric medications within the last six months (per parent report). Further demographic information is provided in Table 1 below.

For the experimental group, ASD classification was based on DSM-5 diagnostic criteria for autism spectrum disorder and confirmed using the ADOS-2 by a trained and research reliable rater. Comparison participants were medically healthy and had no current or prior psychiatric diagnoses. Exclusion criteria included Fragile-X Syndrome, Down Syndrome, traumatic brain injury (TBI), a history of epilepsy or seizures, intellectual disability (IQ less than 75), a contrast-sensitivity impairment, or a visual impairment that could not be corrected with glasses. Comparison participants were excluded from the current study if they had a prior DSM-4 or DSM-5 diagnosis, a history of developmental delays, or a first-degree relative with ASD. Comparison children were also excluded if they scored in the ASD range on CARS-2.

All procedures performed in studies involving human participants were in accordance with the ethical standards of the institutional and/or national research committee and with the 1964 Helsinki declaration and its later amendments or comparable ethical standards. This article does not contain any studies with animals performed by any of the authors. All study procedures were approved by the Teachers College, Columbia University Institutional Review Board. Administration of the SB5 ABIQ and the ADOS-2 were also approved by the Weill Cornell Medical College (WCMC) Institutional Review Board for screening purposes. This part of the testing could have also taken place at the Center for Autism and the Developing Brain at New York-Presbyterian’s Westchester Division. Informed consent was obtained from a parent or legal guardian of all participating children. All participants were monetarily compensated for their participation with an Amazon gift card.

### 2.3. Experimental Stimuli

**Figure 1:**
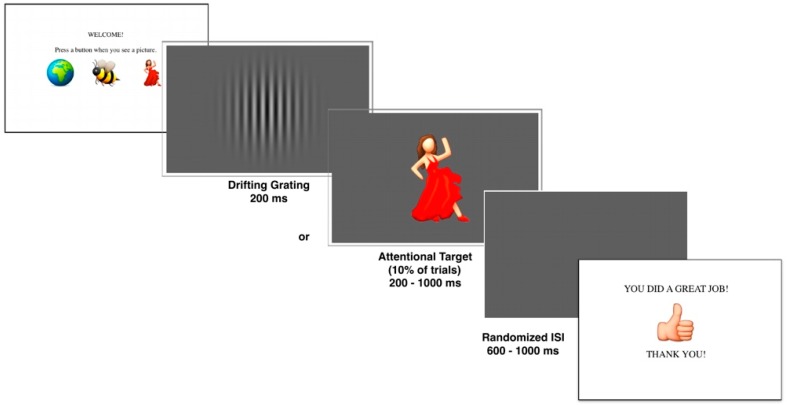
Schematic of the P1 event-related potential task.

The visual task consisted of passively viewing drifting vertical grayscale sinusoidal gratings (Figure 1: drifting grating). Sinusoidal gratings, also known as sine wave gratings, can vary in spatial frequency, orientation, contrast, size, and phase. In the present study, we only manipulated visual contrast and size. The stimuli implemented were similar to those used in behavioral studies of discrimination thresholds, but did not require participants to indicate direction of motion [11,12,30]. Stimuli were programmed using Psykinematix [31] and presented using a 24″ NEC MultiSync PA241W LCD display (1920 × 1200, 60 Hz, 120 cd/m^2^). Stimuli consisted of low-spatial frequency (1 cycle/degree) drifting vertical monochrome gratings surrounded by two-dimensional Gaussian envelopes. Contrast was defined according to the *Michaelson contrast equation* where c = Î∂ with the luminance modulated around the background level. Stimulus size (large vs. small) was either 5.0° or 0.7° and stimulus contrast (bright vs. faint) was either 92% or 2.8%. The stimuli were presented on a uniform gray background so that luminance was consistent across all conditions at 40 cd/m^2^, with only contrast and size varying. There were four conditions of stimuli: Small/High-Contrast Vertical Grayscale Sinusoidal Grating; Large/High-Contrast Vertical Grayscale Sinusoidal Grating; Small/Low-Contrast Vertical Grayscale Sinusoidal Grating; Large/Low-Contrast Vertical Grayscale Sinusoidal Grating.

The four conditions were divided into separate blocks, each consisting of 250 trials. Block order was counterbalanced across participants. Each individual trial began with a blank screen appearing for 150 ms (Figure 1). A grayscale vertical sinusoidal grating of either high (92%) or low (2.8%) contrast and small (0.7°) or large (5.0°) size then appeared in the center of the screen. The vertical sinusoidal gratings drifted either left or right (at 50% chance) at a rate of 2°/s, for 200 ms. Since this was a passive paradigm, to maintain participant attention during the experimental task, participants were instructed to press “1” on the response box when an emoticon appeared as an attentional target (10% of trials per block). If participants did not respond to the attentional task, the experiment continued after 1000 ms. The attentional trials were not included in data analysis and were only to maintain participant interest in the overall passive visual task. Interstimulus interval was randomized, ranging from 600 to 1000 ms. There were four blocks, each consisting of 250 trials, making the total experiment run time approximately 20 min, plus additional time for sensor application and to allow for varying button-press response rates.

### 2.4. Experimental Task

All acquisition of EEG data took place in the Neurocognition of Language Laboratory (NCLLab) at Teachers College, Columbia University. EEG data were recorded continuously from 128 electrode sites throughout the experimental task, in an electrically and sound shielded room at mesopic light levels—a low lighting condition in which both cones and rods contribute to visual processing. The average temperature of the room was 70.9° F and the average humidity of the room was 35.8%. Participants were seated in a wooden chair 116.84 cm away from the monitor and provided with a footrest if necessary. In order to precisely correlate recorded electrical activity from specific locations on each participant’s scalp, 128-channel high-impedance Geodesic Sensor Nets [32] were placed on each participant’s head following measurement of head circumference and marking of the vertex. Each net was soaked a potassium-chloride solution with baby shampoo for five minutes to maximize signal detection and minimize scalp impedances. After the sensor net was applied, individual sensors were adjusted so that individual impedance thresholds were below 40 kΩ. Horizontal eye movements were measured using channels 126 and 127, located at positions to the left and right of the eyes. Channels 8 and 26, positioned above and below the eyes, were used to record horizontal eye saccades. The sensor nets were connected to a high-input amplifier (Net Amps200, Electrical Geodesics Inc., Eugene, OR, USA), and amplified analog voltages were digitalized at a 500 Hz sampling rate. EEG data were referenced to the vertex (Cz) electrode during recording and later re-referenced during analysis (described below). Individual sensors were adjusted prior to beginning the first condition, and midway throughout the experiment, so that impedances were maintained below 40 kΩ throughout all conditions.

### 2.5. Data Analysis

Raw EEG data were pre-processed using NetStation, version 4.5.6 (Electrical Geodesics Inc.), using standard protocols for ERP analysis [33,34,35]. Raw data were digitally filtered offline using a 0.3 to 30 Hz bandpass filter (FIR Passband Gain: 99.0% (−0.1 dB), Stopband Gain: 1.0% (−40.0 dB), Rolloff: 2.00 Hz). Data were subjected to automatic artifact rejection protocols for the removal of movement and physiological artifacts. Bad channels (defined as >150 μV), blink artifacts (defined as >140 μV), and eye saccades (defined as >55 μV) were removed using an in-house NetStation script. Therefore, only trials free of artifacts from participant movements were included in ERP averages for each condition. Data were re-referenced to the average reference, which was calculated by subtracting the mean of all electrodes from each individual channel [36].

**Figure 2:**
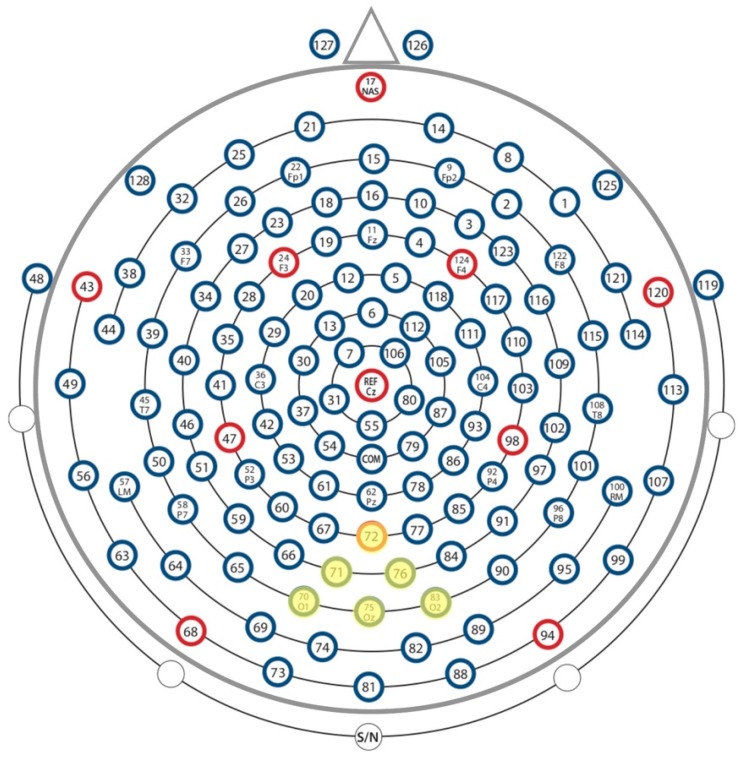
P1 montage of occipital electrodes.

The ERP waveform was segmented into 600 ms epochs, including 200 ms pre-stimulus (baseline) and 400 ms post-stimulus. Individual segments were averaged together for each condition in order to identify time-locked event-related responses associated with the onset of the four conditions (sinusoidal gratings of differing contrast & size). Segments were baseline corrected using the 200 ms before the start of the epoch, when no stimuli were being presented, to provide an average of brain activity unrelated to stimulus processing. Post-processed data files were read into R version 3.1.2 (1.65 Mavericks build). A P1 occipital montage was applied to the data in order to examine evoked responses (Figure 2). P1 peak latency and adaptive mean amplitude for each individual participant were calculated for each of the four conditions, with the P1 peak defined as the greatest positive point between 100 and 200 ms after stimulus presentation. The adaptive mean amplitudes were calculated for each participant and ERP by averaging 10 samples, equivalent to 20 ms, on either side of the peak. The assumptions of equality of variances and normality were tested prior to conducting within- and between-group analyses, using Levene’s statistic and the Shapiro-Wilk test respectively. A one-way analysis of variance (ANOVA) was conducted for the high- and low-contrast experiments separately to test for between-group effects (Size: Small (0.7°), Large (5.0°) × Group: ASD, NT). Non-parametric tests were implemented when the assumption of equality of variances was violated. In order to determine within-group differences, paired-samples *t*-tests were conducted for the high- and low-contrast experiments separately (Small × Large, ASD; Small × Large, NT). For data presentation, individual files were grand-averaged together by group (ASD vs. NT) for each individual condition. P1 grand average ERP waveforms were created in R by averaging conditions across participants.

## 3. Results

### 3.1. Assessment Results

**Figure 3:**
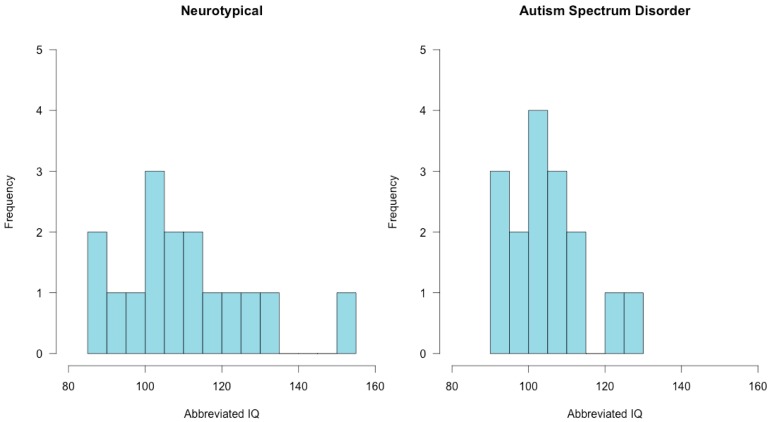
Abbreviated IQ Distribution: Histograms depicting the distribution of abbreviated IQ in neurotypical children (**left**) and children with autism spectrum disorder (**right**).

**Figure 4:**
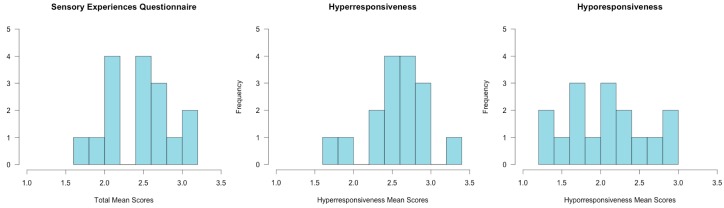
Sensory Experiences Questionnaire Distribution: Histograms depicting the distribution of sensory experiences questionnaire total mean scores, hyperresponsiveness mean scores, and hyporesponsiveness mean scores in children with ASD.

All participants had average or above average ABIQ on the SB5. Abbreviated IQ ranged from 94 to 130 for children with ASD and from 85 to 155 for the neurotypical comparison group. There was no significant difference in ABIQ on the SB5 between children with ASD (*M* = 105, SD = 10.05) and neurotypical children (*M* = 110.56, SD = 17.86; t(30) = −1.02, *p* = 0.31) (Figure 3). ADOS-2 cutoff scores for the experimental group confirmed that all participants were on the autism spectrum. ADOS-2 calibrated severity scores ranged from 6 to 10 (*M* = 7.56, SD = 1.36). Half of the participants in the experimental group (*n* = 8) were categorized as having a high level of autism-spectrum related symptoms, and half (*n* = 8) were categorized as having a moderate level of autism-spectrum related symptoms. None of the comparison participants met criteria for a diagnosis of autism on the CARS-2. In children with ASD, SEQ mean scores ranged from 1.49 to 3.09 (*M* = 2.45, SD = 0.40) with 6 participants classified as in the “typical range”, 5 participants classified as in the “at risk” range, and 5 participants classified as in the “deficient” range. Mean hyporesponsiveness scores ranged from 1.33 to 3.00 (*M* = 2.07, SD = 0.52), and mean hyper-responsiveness scores ranged from 1.78 to 3.35 (*M* = 2.57, SD = 0.40) in the experimental group (Figure 4). Based on SEQ cut-off scores, within the experimental group, 6 participants were classified as hyper-responsive, 1 participant was classified as hyporesponsive, 8 participants were classified as being both hyper- and hyporesponsive, and 1 participant was classified as having no pattern of responsiveness to sensory stimuli. Among the neurotypical comparison participants, mean scores ranged from 1.00 to 2.87 (*M* = 1.53, SD = 0.44), with 15 participants classified as in the “typical range” and 1 participant classified as being in the “deficient” range. Mean hyporesponsiveness scores ranged from 1.00 to 2.83 (*M* = 1.40, SD = 0.49), and hyperresponsiveness scores ranged from 1.00 to 2.78 (*M* = 1.55, SD = 0.45) in the neurotypical comparison group. One participant from the neurotypical comparison group was classified as being hyper- and hyporesponsive to sensory stimuli, and the remaining 15 participants from the comparison group showed no specific pattern of responsiveness.

### 3.2. EEG Results

Following data pre- and post-processing, numbers of usable trials were documented for each participant. A two-way ANOVA was conducted to examine the effect of group (ASD, NT) and condition (Small, Large) on number of usable trials in the high-contrast experiment. Although children with ASD had fewer usable trials per condition (Small, High Contrast *M* = 96.87, SD = 51.93; Large, High Contrast *M* = 98.50, SD = 51.77) than NT comparison children (Small, High Contrast *M* = 125.68, SD = 54.68; Large, High Contrast *M* = 125.62, SD = 57.43), this difference was not statistically significant (*F*(1, 60) = 0.01, *p* = 0.90). The same two-way ANOVA analysis was also conducted to examine the effect of group (ASD, NT) and condition (Small, Large) on number of usable trials in the low-contrast experiment. Although children with ASD had fewer usable trials per condition (Small, Low-Contrast *M* = 97.50, SD = 55.10; Large, Low-Contrast *M* = 98.07, SD = 54.28) than NT comparison children (Small, Low-Contrast *M* = 148.07, SD = 41.23; Large, Low-Contrast *M* = 142.78, SD = 52.31), this difference was also not statistically significant (*F*(1, 52) = 0.16, *p* = 0.68). The threshold for inclusion based on usable trials per condition was set at 40 usable trials per condition.

## 4. High-Contrast Experiment

### 4.1. Peak Latency Analyses

**Figure 5:**
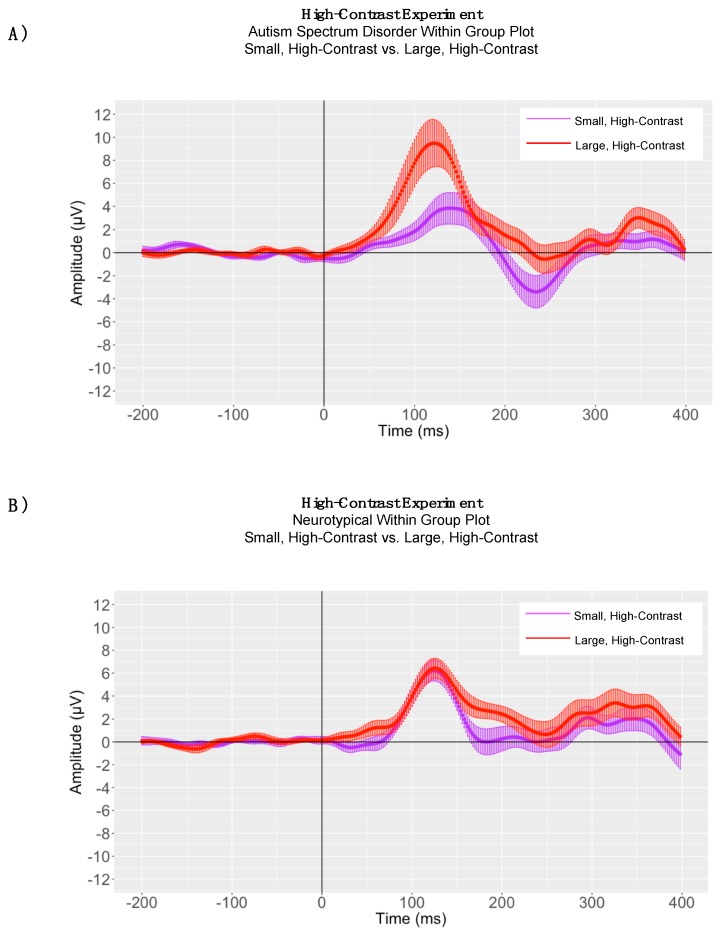
High-Contrast Experiment: (**Panel A**) Autism Spectrum Disorder Grand Average ERP Plot and (**Panel B**) Neurotypical Grand Average ERP Plot, where the solid lines represent the grand averaged waveform to small, high-contrast drifting low-frequency sinusoidal gratings (purple) and large, high-contrast drifting low-frequency sinusoidal gratings (red) and the shaded lines represent the standard error of the mean amplitude at each time point.

Within Group Analyses: Paired samples *t*-tests were conducted separately to evaluate the significance of P1 peak latency differences in processing small, high-contrast sinusoidal gratings versus large, high-contrast gratings for children with ASD and neurotypical comparison children, separately. As predicted, in the high-contrast experiment participants with ASD demonstrated shorter P1 latencies to large, high-contrast gratings (5.0°, 98% contrast) compared to small, high-contrast gratings (0.7°, 98% contrast) (large, high-contrast P1 mean peak latency = 125.69, SD = 15.43; small, high-contrast P1 mean peak latency = 142.38; SD = 26.68) (P1: t(15) = 2.55, *p* = 0.022, d = 0.63; (Figure 5, Panel A). Neurotypical comparison participants demonstrated no statistically significant difference in P1 latency between large, high-contrast gratings compared to small, high-contrast gratings (large, high-contrast P1 mean peak latency = 131.37 ms, SD = 17.06; small, high-contrast P1 mean peak latency = 126.50, SD = 13.07); *p* = 0.35 (Figure 5, Panel B).

**Figure 6:**
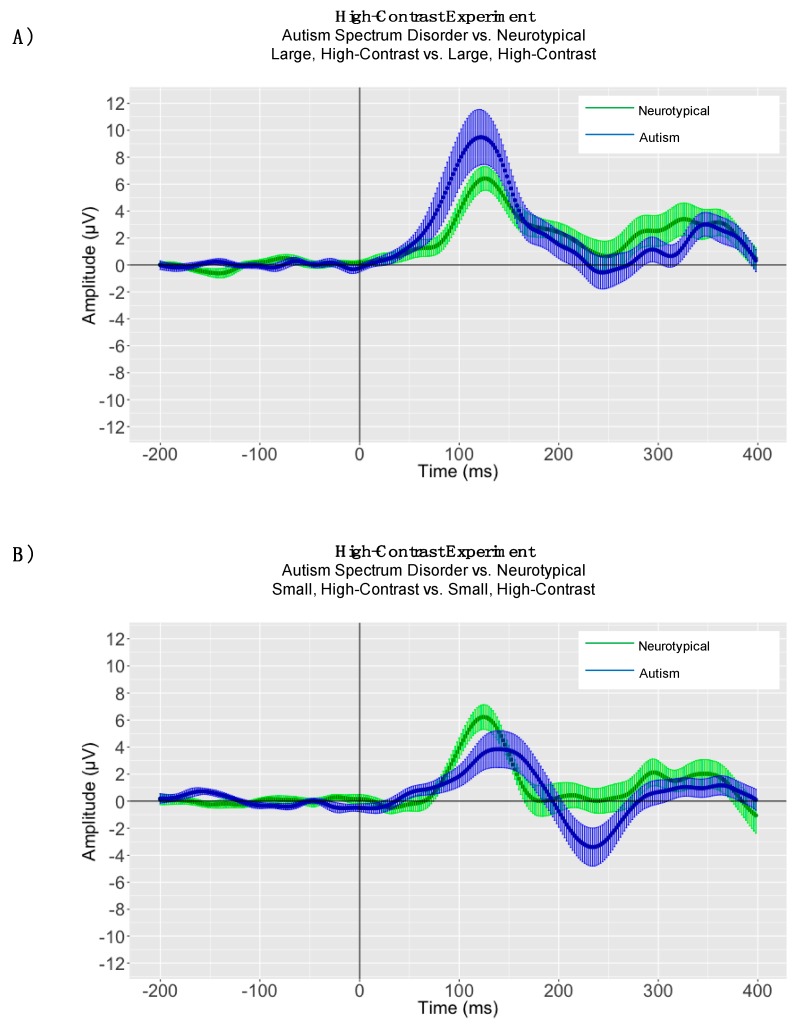
High-Contrast Experiment: (**Panel A**) Autism Spectrum Disorder vs. Neurotypical Grand Average ERP Plot where the solid lines represent the grand averaged waveform to large, high-contrast drifting low-frequency sinusoidal gratings in children with autism (blue) and neurotypical children (green). The shaded lines represent the standard error of the mean amplitude at each time point. (**Panel B**) Autism Spectrum Disorder vs. Neurotypical Grand Average ERP Plot where the solid lines represent the grand averaged waveform to small, high-contrast drifting low-frequency sinusoidal gratings in children with autism (blue) and neurotypical children (green). The shaded lines represent the standard error of the mean amplitude at each time point.

Between-Group Analyses: A one-way analysis of variance (ANOVA) was conducted to evaluate the significance of P1 latency differences in processing large, high-contrast gratings between children with ASD and neurotypical comparison children. In the high-contrast experiment, there was no significant between-group difference in large, high-contrast P1 latency (ASD: P1 mean peak latency = 125.69 ms, SD = 15.43; NT: P1 mean peak latency = 131.38 ms, SD = 17.06, *p* = 0.25) (Figure 6, Panel A). Although latencies were normally distributed for both children with ASD and neurotypical children in the small, high-contrast condition (*p’s* > 0.05), the assumption of homogeneity of variances was violated, as assessed by Levene’s test for equality of variances (P1: *p* = 0.008). Therefore, a Mann-Whitney U test was run to determine the significance of differences in processing small, high-contrast gratings between children with ASD and neurotypical comparison children. The distributions of P1 latencies for children with ASD and neurotypical comparison children were not similar, as assessed by visual inspection. For the small, high-contrast condition there was no statistically significant difference in P1 latency between children with ASD and neurotypical children (ASD: P1 mean peak latency = 142.38 ms, SD = 26.68; NT: P1 mean peak latency = 126.50 ms, SD = 13.07, *p* = 0.08) (Figure 6, Panel B).

### 4.2. High-Contrast Adaptive Mean Amplitude Analyses

In children with autism, P1 adaptive mean amplitudes were larger in magnitude for large, high-contrast gratings (P1 mean = 11.80 μV, SD = 7.50) than for small, high-contrast gratings (P1 mean = 5.06 μV, SD = 4.04); (P1: t(15) = −4.80, *p* < 0.0001) (Figure 5). In neurotypical children there was no significant difference in P1 adaptive mean amplitudes between large, high-contrast gratings (P1 mean = 6.46 μV, SD = 3.33) and small, high-contrast gratings (P1 mean = 5.92, SD = 3.31); (P1: t(15) = −0.45, *p* = 0.65) (Figure 5). When conducting between-group analyses for the large, high-contrast condition, the assumption of homogeneity of variances was violated, as assessed by Levene’s test for equality of variances (P1: *p* = 0.009). Therefore, a Mann-Whitney U test was run to determine between-group differences in processing large, high-contrast gratings. P1 adaptive mean amplitude for large, high-contrast gratings was larger in magnitude for children with autism (P1 mean = 11.80 μV, SD = 7.50, mean rank = 20.19) than for neurotypical children (P1 mean = 6.46 μV, SD = 3.33, mean rank = 12.81), U = 69.00, *p* = 0.02. However, for the small, high-contrast grating there was no statistically significant difference between children with autism spectrum disorder and neurotypical children in P1 adaptive mean amplitude (P1: *p* = 0.41).

### 4.3. Electrophysiological Associations with Cognitive and Behavioral Measures

**Figure 7:**
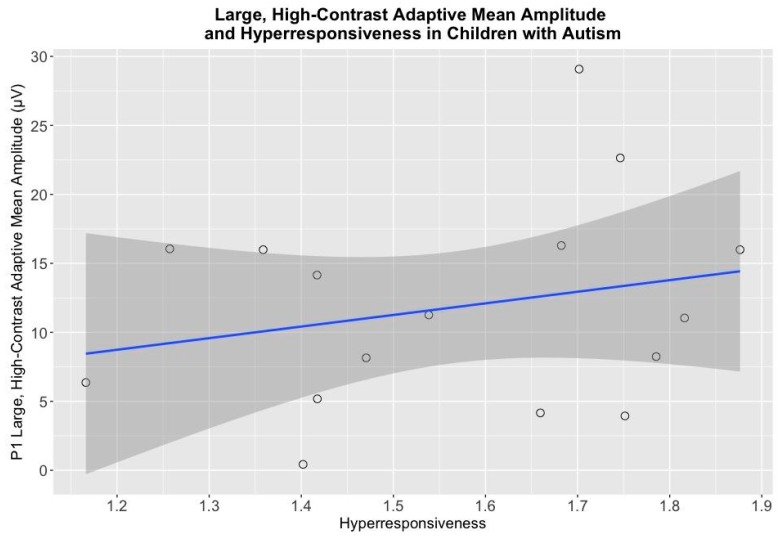
Large, High-Contrast P1 Adaptive Mean Amplitude and Hyperresponsiveness in Children with Autism: Scatterplot depicting mean hyperresponsiveness scores on the *x*-axis and P1 adaptive mean amplitude to large, high-contrast drifting low-frequency sinusoidal gratings on the *y*-axis.

A linear regression established that when controlling for chronological age, P1 adaptive mean amplitude for large, high-contrast gratings significantly predicted hyperresponsiveness item mean scores on the SEQ (P1: *F*(2, 13) = 4.42, *p* < 0.05) (Figure 7). Adaptive mean amplitude for large, high-contrast gratings accounted for 40% (P1) of the variation in hyperresponsiveness item mean scores on the SEQ with an adjusted R^2^ = 0.31 for P1, a medium effect size according to Cohen (1988). There was no relationship between hypo- or hyperresponsiveness and adaptive mean amplitude for small or large gratings for NT children or for small gratings for children with autism (*p’s* > 0.05).

## 5. Low-Contrast Experiment

Since the threshold for useable trials per condition was set at 40, two participants from the experimental group and two participants from the comparison group were excluded from analysis for low-contrast conditions due to <40 usable trials per condition. After exclusions, 28 participants were included in data analysis for the low-contrast experiment (ASD: 14; NT: 14). Although the ASD group after exclusions was younger on average than the NT group after exclusions (ASD mean age = 8.32 years, SD = 1.72; NT mean age = 9.47 years, SD = 2.10), there was still no significant difference in chronological age between the two groups (t(1, 26) = 2.46, *p* = 0.12).

### 5.1. Within-Group Peak Latency Analyses

**Figure 8:**
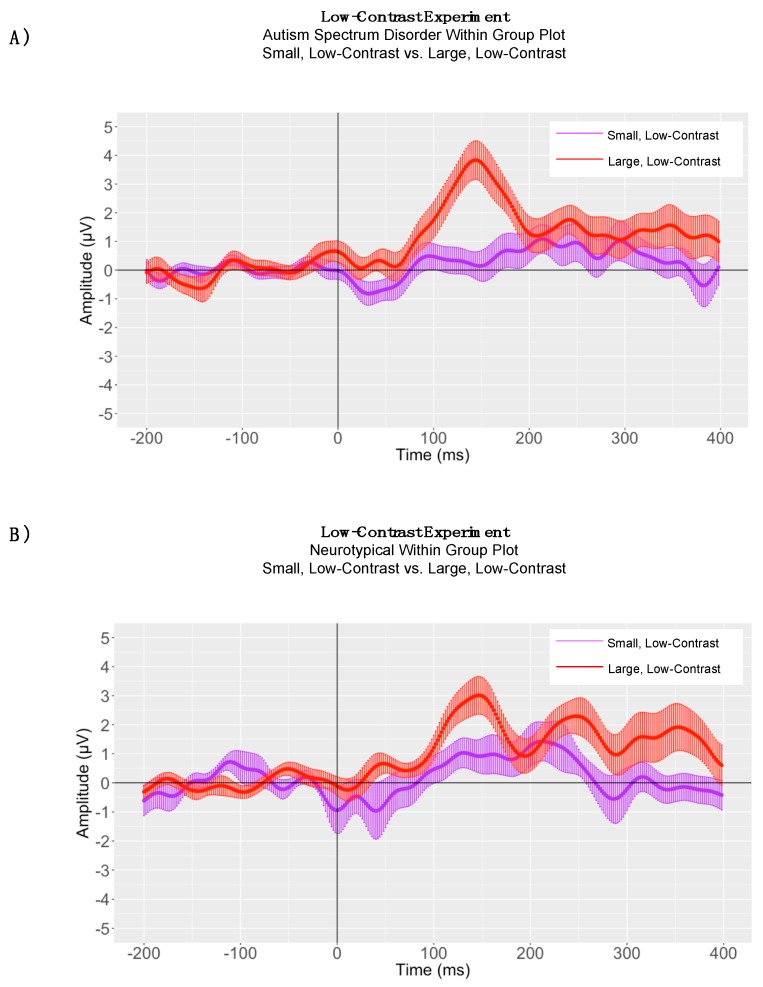
Low-Contrast Experiment: (**Panel A**) Autism Spectrum Disorder Grand Average ERP Plot and (**Panel B**) Neurotypical Grand Average ERP Plot, where the solid lines represent the grand averaged waveform to small, low-contrast gratings (in purple) and large, low-contrast gratings (in red) and the shaded lines represent the standard error of the mean amplitude at each time point.

Paired samples *t*-tests were conducted to evaluate the significance of P1 peak latency differences in processing small, low-contrast gratings versus large, low-contrast gratings for children with ASD and neurotypical comparison children, separately. In the low-contrast condition, there were no significant P1 latency differences between small and large gratings for children with ASD (small grating P1 mean peak latency = 151.43, SD = 37.84; large grating P1 mean peak latency = 153.00 ms, SD = 28.08; t(13) = −0.10, *p* = 0.91) (Figure 8, Panel A). When conducting within-group analyses for P1 responses in neurotypical children, the assumption of homogeneity of variances was violated, as assessed by Levene’s test for equality of variances (P1: *p* = 0.04). Therefore, a Mann-Whitney U test was run. In neurotypical children, there were no significant P1 latency differences between small and large gratings (small grating N1 mean peak latency = 144.14, SD = 37.35, mean rank = 13.93; large grating N1 mean peak latency = 144.14 ms, SD = 24.28, mean rank 15.07; U = 106, *p* = 0.73) (Figure 8, Panel B).

### 5.2. Between-Group Peak Latency Analyses

**Figure 9:**
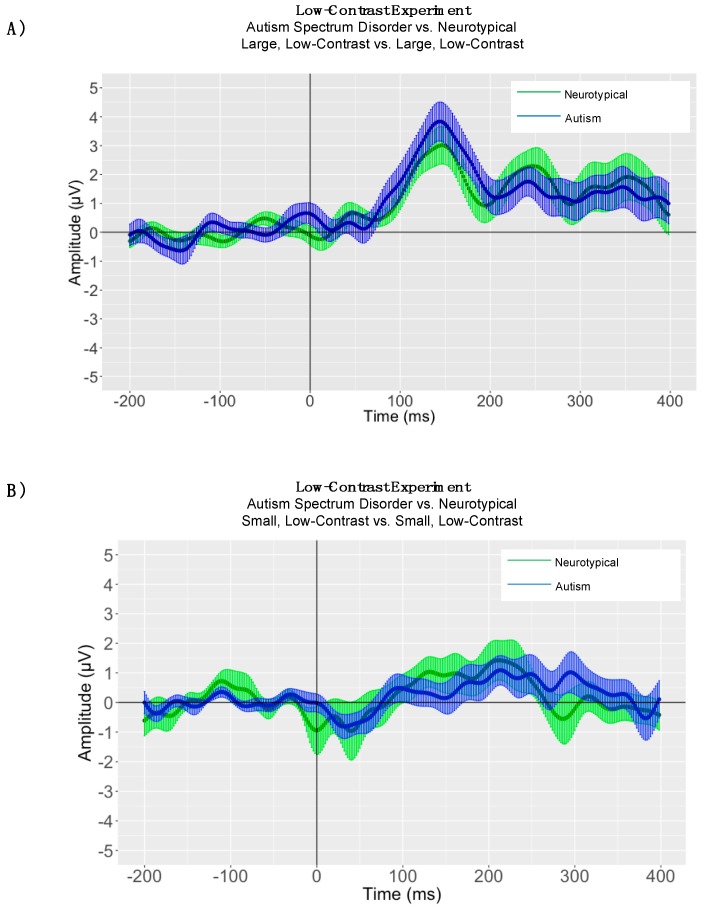
Low-Contrast Experiment: (**Panel A**) Autism Spectrum Disorder vs. Neurotypical Grand Average ERP Plot where the solid lines represent the grand averaged waveform to large, high-contrast gratings in children with autism (blue) and neurotypical children (green). The shaded lines represent the standard error of the mean amplitude at each time point. (**Panel B**) Autism Spectrum Disorder vs. Neurotypical Grand Average ERP Plot where the solid lines represent the grand averaged waveform to small, high-contrast gratings in children with autism (blue) and neurotypical children (green). The shaded lines represent the standard error of the mean amplitude at each time point.

One-way ANOVAs were conducted to evaluate the significance of P1 latency differencesin processing large, low-contrast gratings between children with ASD and neurotypical comparison children. In the large, low-contrast condition, there were no significant P1 latency differences between children with ASD (P1 mean peak latency = 153.00, SD = 28.08) and NT comparison children (P1 mean peak latency = 144.14; SD = 24.28; P1: *F*(1, 26) = 0.79, *p* = 0.38) (Figure 9, Panel A). In the small, low-contrast condition no significant P1 latency differences were found between children with ASD (P1 mean peak latency = 151.43, SD = 37.84) and NT comparison children (P1 mean peak latency = 144.14, SD = 37.35; P1: *F*(1, 26) = 0.26, *p* = 0.61) (Figure 9, Panel B).

### 5.3. Adaptive Mean Amplitude Analysis

P1 adaptive mean amplitude was larger in magnitude for large, low-contrast gratings (P1 mean = 3.85 μV, SD = 2.17) than for small, low-contrast gratings (P1 mean = 1.30 μV, SD = 1.61) for children with autism (P1: t(13) = −4.89, *p* < 0.0001). There was no significant difference in P1 adaptive mean amplitudes between large, low-contrast (P1 mean = 3.22 μV, SD = 2.13) and small, low-contrast gratings (P1 mean = 1.89 μV, SD = 2.55) for neurotypical children (P1: t(13) = −1.70, *p* = 0.11) (Figure 8). There was also no statistically significant difference between children with autism and neurotypical children in P1 adaptive mean amplitude for large, low-contrast gratings as determined by a one-way ANOVA (ASD P1 mean = 3.85 μV, SD = 2.17; NT P1 mean = 3.22 μV, SD = 2.13); (P1: *F*(1, 26) = 0.60). Additionally, there was no statistically significant difference between children with autism and neurotypical children in P1 adaptive mean amplitude for small, low-contrast gratings (ASD P1 mean = 1.30 μV, SD = 1.61; NT P1 mean = 1.89 μV, SD = 2.55); (P1: *F*(1, 26) = 0.51, *p* = 0.47) (Figure 9).

## 6. Study Limitations and Delimitations

This study has a number of limitations that we plan to take into consideration when implementing future studies. First and foremost, ASD is a heterogeneous neurodevelopmental disorder and we had a relatively small sample size with restrictive inclusion and exclusion criteria which resulted in a group of medication-free children with autism with average or above-average intellectual ability. Additionally, the more-able group of children with autism that participated in the present study represented a wide range of sensory processing profiles, based on the SEQ, that we were unable to fully explore due to our restricted sample size. Another limitation was the lack of sensitivity in utilizing the SEQ for identifying subgroups based on sensory responsiveness. In the present study *n* = 8 children with autism were categorized as being both hyper- and hyporesponsive to sensory stimuli based on SEQ cut-off scores for each pattern of responsiveness. Therefore, the SEQ was not a sensitive enough measure to adequately categorize the sensory profiles present in this population. Future studies should attempt to utilize more comprehensive sensory questionnaires that can provide information regarding responsiveness within specific sensory domains. A more sensitive categorization of sensory responsiveness would allow for researchers to sub-group based on hyper- or hyporesponsiveness patterns and identify if there are any electrophysiological signatures associated with specific patterns of sensory responsiveness.

Although our task was similar to that used by previous researchers [11,12,30] we implemented a passive paradigm and did not require participants to indicate the perceived direction of motion during the ERP task. Rather, participants were instructed to focus on the center of the screen at the lines moving left or right and to respond to emoticons with a button press to maintain engagement in the experiment. We utilized ERP latency and adaptive mean amplitude to make inferences about motion processing instead of discrimination thresholds anticipating that many young children with ASD may not be able to follow directions with a high degree of reliability. Additionally, in the present study we only manipulated visual contrast and the size of the gratings. Since spatial suppression is a non-linear sensory effect, future studies should consider implementing additional sizes and spatial frequencies to thoroughly examine contrast sensitivity function in autism.

There is evidence to support that the magnocellular pathway, which is responsible for motion detection, undergoes developmental maturation through middle childhood [37,38]. Therefore, prospective studies should consider enrolling individuals within a tighter age range to reduce the potential variability introduced by developmental changes in motion processing. Lastly, due to the inconsistent prior findings related to low-level visual processing in autism [39], these findings should be replicated in order to further elucidate spatial suppression and contrast gain differences in individuals with ASD and their potential downstream effects on sensory processing.

## 7. Discussion

In the present study, children with autism displayed enhanced sensitivity to large, high-contrast low-spatial frequency stimuli as indexed by significantly shorter P1 response latencies to large vs. small gratings (see Table 2 for a results summary). Alternatively, this within-group finding could reflect delayed processing of small, high-contrast drifting gratings. This is consistent with behavioral findings of decreased discrimination thresholds in conditions of high-contrast and increased discrimination thresholds to small stimuli [11,13] and could potentially underlie altered lateral inhibitory mechanisms. Shorter P1 latencies in conditions of large, high-contrast stimuli for children with ASD could also lend support to the excitatory/inhibitory (E/I) imbalance theory of ASD, since weakened neural inhibition or increased neural excitation could result in a diminished spatial suppression effect resulting in enhanced processing of large, high-contrast stimuli [40]. If the P1 ERP were to be utilized as a marker of suppression to visual motion, we would have expected controls to display longer latencies to large, high-contrast gratings compared to small, high-contrast gratings. Although neurotypical participants did demonstrate longer P1 latencies to large, high-contrast gratings than small, high-contrast gratings, this comparison did not reach statistical significance. This is likely attributable to our analysis being underpowered due to small sample size and the within-group variability in latency of response potentially due to maturational changes in the magnocellular pathway. In the low-contrast experiment, as expected we found no significant within-group or between-group latency differences for neurotypical children since contrast gain is primarily operating at this level. We also found no significant within-group differences for children with autism.

The current study found children with autism had larger amplitude responses to large gratings irrespective of contrast, which is suggestive of differences in both visual suppression and contrast gain control in autism. Additionally, children with autism had larger P1 adaptive mean amplitude responses to the large, high-contrast condition as compared to neurotypical children. A prior study that examined ERP responses to the expansion of radial rings found that more-able adults with autism demonstrated significantly larger motion-onset N170 peak amplitudes than controls, but found no differences in P1 [41]. Taken together, these findings suggest differences in motion processing persist across development in autism. In the present study, an N170 response was not consistently observed at occipital electrode sites, but children with autism displayed a negative deflection to small, high-contrast stimuli at approximately 200 ms post-stimulus presentation. This could potentially reflect a response to motion offset, but this effect would need to be investigated in an independent sample. We also found that P1 adaptive mean amplitude in response to large, high-contrast sinusoidal gratings significantly predicted hyperresponsiveness item mean scores on the sensory experiences questionnaire for children with autism. In other words, children with the highest levels of hyperresponsiveness had the smallest P1 adaptive mean amplitudes. Normal contrast sensitivity function is dependent on intact contrast gain control [42]; therefore, it is possible that hyperresponsiveness to sensory stimuli could reflect deficits in contrast gain control.

Cortical processing of visual stimuli is not considered to be entirely hierarchical since feedforward connections have corresponding reverse feedback connections, which modulate responses to stimuli within a receptive field [20]. Spatial attention is mediated by feedback interactions in the parietal cortex or feedforward connections via the thalamus. Both feedforward and feedback connections are primarily excitatory and are thought to play a role in attention and visual awareness [20]. Bullier et al. (1996) inactivated V2 with a GABA injection in non-human primates while recording from V1 and found a reduction in the strength of response to stimuli within the V1 receptive field. However, when presenting to the surround of the receptive field, they unexpectedly found a strong response in the V1 cells [43]. V1, V2, and V3 are dependent on feedback from the middle temporal visual area (MT) and inactivation of this area results in a reduction in surround suppression [44]. A recent study also demonstrated that individuals with autism had atypically large populations of receptive fields in MT [13]. Therefore, it is possible that individuals with autism have functional differences in these projections, which in turn may lead to altered spatial suppression and contrast gain.

The current study provided insights into cortical activity related to a visual motion task in children with ASD. We demonstrated P1 ERP latency and adaptive mean amplitude differences to luminance defined low-spatial frequency drifting gratings in children with autism. To our knowledge this is the first ERP study to investigate whether P1 ERP latency and amplitude can be used to examine low-level visual motion and contrast processing in children with autism. Further identification of differences in low-level visual processing in autism may offer a window into understanding the downstream effects on sensory processing in autism.

## Figures and Tables

**Table 1:** Participant Demographic Information.

	Autism (*n* = 16)	Neurotypical (*n* = 16)	Total (*n* = 32)
*Sex*			
Male	11	9	20
Female	5	7	12
Age in years: Mean (SD)	8.04 (1.78)	9.44 (2.29)	8.94 (2.14)
*Race*			
African-American	4	2	6
Asian-American	1	5	6
Caucasian	6	9	15
Native American	1	0	1
Mixed Race	4	0	4
*Ethnicity*			
Hispanic or Latino	7	2	9
Not Hispanic or Latino	9	14	23

**Table 2:** Results Summary.

	P1 Peak Latency	P1 Adaptive Mean Amplitude
***High-Contrast Experiment***	ASD within group: ***shorter*** latencies to large vs. small. Neurotypical no within group differences. No between group differences.	ASD within group: ***larger*** amplitudes to large vs. small. Neurotypical no within group differences. ASD between group: ***larger*** amplitudes to large gratings than neurotypical group. No between group differences to small gratings. ASD group: amplitudes to large gratings correlated with hyperresponsiveness scores on the SEQ. Neurotypical group: no correlation with SEQ.
***Low-Contrast Experiment***	ASD within group: no differences. Neurotypical within group: no differences. No between group differences.	ASD within group: ***larger*** amplitudes to large gratings vs. small gratings. Neurotypical: no within group differences. No between group differences.
